# Notoginsenoside R1 attenuates ischemic stroke via CKLF1-dependent suppression of neuronal metabolic reprogramming

**DOI:** 10.3389/fphar.2026.1964218

**Published:** 2026-10-19

**Authors:** Hanlong Wang, Shasha Liu, Chen Chen, Junpeng Long, Qian Yan, Yuting Lin, Shuqin Li, Yanting Huang, Songwei Yang, Meiyu Lin, Xuan Liu, Fan Yi, Yong Tan, Naihong Chen, Qidi Ai, Yantao Yang

**Affiliations:** 1 School of Pharmacy, Hunan Engineering Technology Center of Standardization and Function of Chinese Herbal Decoction Pieces, Hunan University of Chinese Medicine, Changsha, China; 2 School of Chinese Materia Medica, Guangzhou University of Chinese Medicine, Guangzhou, Guangdong, China; 3 Department of Pharmacy, Changsha Hospital for Maternal & Child Healthcare Affiliated to Hunan Normal University, Changsha, China; 4 Department of Pharmacy, The First Hospital of Lanzhou University, Lanzhou, China; 5 Key Laboratory of Cosmetic, China National Light Industry, Beijing Technology and Business University, Beijing, China; 6 Nephrology Department, Xiangtan Central Hospital, Xiangtan, China; 7 State Key Laboratory of Bioactive Substances and Functions of Natural Medicines, Institute of Materia Medica & Neuroscience Center, Chinese Academy of Medical Sciences and Peking Union Medical College, Beijing, China; 8 National Key Laboratory Cultivation Base of Chinese Medicinal Powder & Innovative Medicinal Jointly Established By Province and Ministry, Technology Innovation Center, Hunan University of Chinese Medicine, Changsha, China

**Keywords:** chemokine-like factor 1, ischemic stroke, mechanism research, metabolic reprogramming, notoginsenoside R1

## Abstract

**Background:**

Ischemic stroke, a leading cause of global mortality and disability, results from cerebral blood flow disruption, driving metabolic and neuronal dysfunction. Current therapies-thrombolysis, thrombectomy, and neuroprotectants-are constrained by narrow time windows and hemorrhagic risks. Notoginsenoside R1 (NGR1), a bioactive saponin from Panax notoginseng, exhibits multimodal neuroprotection, yet its mechanistic interplay with metabolic pathways remains unclear.

**Objective:**

We investigated NGR1’s neuroprotective efficacy in ischemic stroke, focusing on its modulation of the CKLF1/CCR5 axis and metabolic reprogramming.

**Methods:**

This study employs a multi-tiered approach integrating *in vitro* experiments, *in vivo* animal research, computationally validated target simulations, and gene knockout techniques to systematically elucidate NGR1’s regulatory role in key indicators during ischemic stroke treatment. Molecular docking and kinetic modelling first predicted NGR1-CKLF1 binding, subsequently validated via Cellular Thermal Shift Assay (CETSA) and Drug Affinity Responsive Target Stability (DARTS) experiments. *In vitro* experiments employed a PC12 cell oxygen-glucose deprivation (OGD) model to evaluate NGR1’s neuroprotective effects. CCK-8 assays, live-dead Cell staining, metabolic assay kits, TUNEL and JC-1 staining, Western blot (WB) analysis, and immunofluorescence (IF) co-localization revealed *in vitro* metabolic reprogramming mechanisms. *In vivo* experiments employed a permanent middle cerebral artery occlusion (pMCAO) model to mimic human ischemic pathophysiology. Neuronal metabolic reprogramming mechanisms following NGR1 treatment were elucidated via TTC staining, neurobehavioral testing, Nissl staining, Enzyme-linked immunosorbent assay (ELISA) assays, immunohistochemical (IHC), WB, IF, and Co-immunoprecipitation (Co-IP). Pharmacodynamic evaluation in the CKLF1−/− rat middle cerebral artery occlusion model, employing ELISA assays and WB analysis, confirmed NGR1’s specificity towards CKLF1.

**Results:**

Docking and MD predicted a stable NGR1–CKLF1 interaction; CETSA/DARTS supported a direct physical interaction, but binding-affinity constants were not determined. *In vitro*, NGR1 mitigated OGD-induced metabolic dysregulation, inhibited apoptosis, and enhanced neuronal survival. *In vivo* experiments demonstrated that NGR1 reduced infarct volume, improved neurological behavior scores, mitigated Nissl body damage, and alleviated inflammatory responses. Mechanistically, NGR1 inhibits downstream AMPK/mTOR/HIF-1α-driven neuronal metabolic reprogramming characterized by glycolytic activation by disrupting CKLF1-CCR5 binding. CKLF1 knockout abolished NGR1’s therapeutic effects and its role in neuronal metabolic reprogramming, confirming CKLF1 dependency.

**Conclusion:**

NGR1 alleviates ischemic neuronal injury, at least in part, by interfering with CKLF1/CCR5-associated signaling, thereby attenuating pathological metabolic reprogramming, neuroinflammation, and apoptosis. Clinical relevance requires further validation.

## Graphical Abstract

**Figure:**
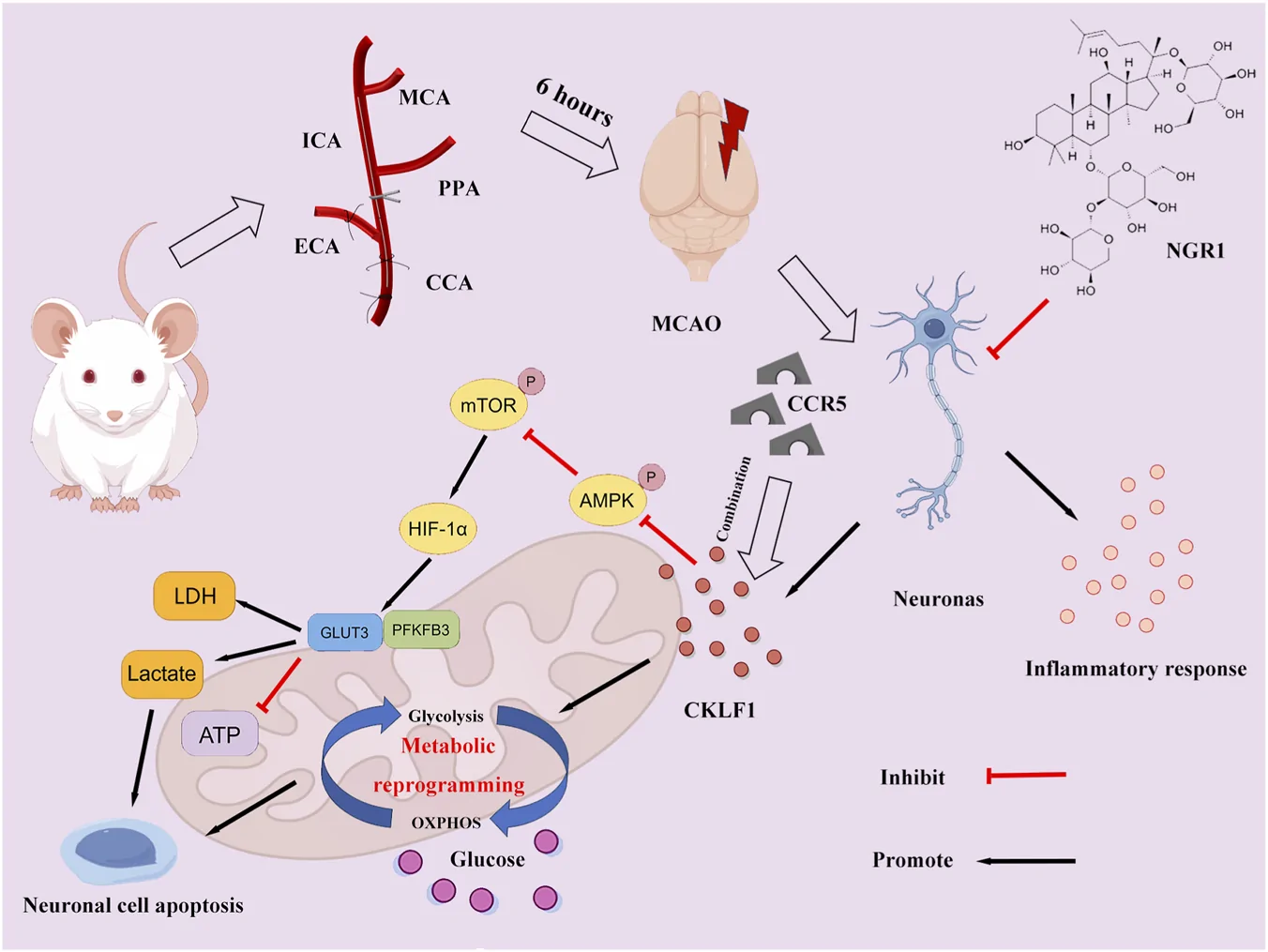

## 1 Introduction

Ischemic stroke arises from disrupted cerebral blood perfusion, triggering focal cerebral ischemia–hypoxia and subsequent neuronal death; it accounts for approximately 80% of all stroke cases (Hankey, 2006). Cerebral vascular thrombosis constitutes its primary pathological cause, which obstructs intracranial blood flow (Ekker et al., 2023). Patients commonly suffer persistent neurological deficits including hemiparesis, aphasia and cognitive dysfunction, with severe cases posing life-threatening risks (Kong et al., 2023). Intravenous thrombolysis with recombinant tissue-type plasminogen activator (rtPA) serves as the first-line acute treatment, yet its therapeutic window is limited to 4.5 h after symptom onset (Jean LeBlanc et al., 2019). Mechanical thrombectomy represents another mainstream interventional approach but carries inherent risks of hemorrhage and reperfusion injury. Edaravone, a clinical neuroprotectant, is also administered to alleviate ischemic brain damage (Xu et al., 2021). Cerebral ischemia provokes oxygen–glucose deprivation (OGD), depleting glucose—the predominant cerebral energy substrate—and disrupting central nervous system (CNS) homeostasis. Restricted cerebral oxygen and glucose delivery suppresses oxidative phosphorylation, forcing neurons to undergo metabolic reprogramming toward enhanced glycolysis to sustain ATP production (Geng et al., 2018; Guo et al., 2021). Though glycolysis generates ATP independent of oxygen via glucose-to-pyruvate conversion (Yu et al., 2025), Neurons switch to inefficient anaerobic glycolysis under ischemia to maintain ATP supply, which triggers massive lactate buildup and subsequent intracellular acidification. Lactate-derived acidosis impairs cellular integrity, disrupts blood–brain barrier (BBB) permeability, and ultimately accelerates neuronal death (Bauernfeind et al., 2014; Zhang et al., 2022). Preclinical evidence demonstrates that inhibiting glycolysis or lactate dehydrogenase A (LDHA) curtails lactate overproduction, relieves cerebral acidosis and reduces infarct volume (Xiong et al., 2024). Accordingly, correcting pathological glycolytic overactivation has emerged as a promising therapeutic strategy to preserve neuronal viability after ischemia.

Chemokine-like factor 1 (CKLF1), a non-classical CC chemokine-like factor discovered in 2001, is a hydrophobic secreted protein existing in at least three isoforms. Its primary receptor, C-C chemokine receptor 5 (CCR5), is a well-established therapeutic target in ischemic stroke (Joy et al., 2019; Chen et al., 2020). CKLF1 exhibits distinct spatiotemporal expression patterns: it is abundant in the fetal brain but barely detectable in intact adult brain tissue, while cerebral ischemia markedly upregulates neuronal CKLF1 expression, implying dual roles in neurodevelopment and ischemic injury (Kong et al., 2011; Wang et al., 2012). Recent studies highlight the CKLF1/CCR5 axis as a key modulator of post-stroke neuroinflammation and metabolic reprogramming. In microglia, CKLF1 shifts cellular metabolism toward glycolysis by activating the AMPK/mTOR/HIF-1α cascade, driving pro-inflammatory polarization and impaired phagocytosis (Ma et al., 2023). Ischemia-induced neuronal CCR5 upregulation synergizes with elevated CKLF1 to amplify neuroinflammatory signaling, positioning the CKLF1/CCR5 pathway as a critical bridge linking metabolic dysfunction and immune activation in ischemic brain lesions. Targeting CKLF1/CCR5 signaling may simultaneously mitigate ischemia-triggered glycolytic dysregulation, neuroinflammation and neuronal apoptosis to facilitate functional recovery.

Sanchi (*Panax notoginseng* (Burk.) F. H. Chen), the dried root and rhizome of Araliaceae medicinal herb, is a classical materia medica in traditional Chinese medicine (TCM). It is sweet and slightly bitter in flavor, warm in nature, and tropism to the liver and stomach meridians. Its canonical therapeutic effects are dissipating blood stasis to arrest bleeding, reduce swelling and relieve pain. Under TCM theory, cerebral ischemic stroke falls under the category of apoplexy caused by blood stasis obstructing cerebral collaterals; for thousands of years, Sanchi has been widely prescribed to treat cerebrovascular disorders by activating cerebral blood circulation and unblocking stagnant collaterals. Clinical data further validate its safety and therapeutic efficacy against acute ischemic stroke (Chen et al., 2008).

Modern pharmacological research confirms that Panax notoginseng saponins (PNS) alleviate ischemic pathological progression via antiplatelet activity and improve neurological outcomes in stroke patients (Zhang et al., 2026). Notoginsenoside R1 (NGR1), a characteristic protopanaxatriol-type saponin isolated from PNS, exerts robust neuroprotective, anti-inflammatory and metabolism-regulating effects (Lin et al., 2026). Preclinical animal studies have verified that NGR1 (10–40 mg/kg) reduces infarct size, attenuates BBB breakdown and restores neuronal energy homeostasis by boosting glucose uptake and mitochondrial ATP synthesis (Liu et al., 2021; Liu B. et al., 2022). Previous studies of CKLF-mediated metabolic remodeling focus largely on microglia, with its neuronal glycolysis-regulating function uncharacterized. Although NGR1 exerts multifaceted neuroprotection, its direct targets remain elusive and impede translational progress. Here, we verified CKLF1 as a direct binding partner of NGR1 via molecular docking and cellular/animal assays. NGR1 suppresses CKLF1-initiated neuronal metabolic reprogramming to restore energy homeostasis and mitigate ischemic brain injury, establishing a new neuroprotective mechanism.

## 2 Materials and methods

### 2.1 Materials

NGR1 (molecular formula: C_47_H_80_O_18_; molecular weight: 933.14 Da; purity ≥98%) was purchased from Shanghai Ronghe Pharmaceutical Technology Development Co., Ltd (Shanghai, China) PC12 cell line with high differentiation status was obtained from Guangzhou Cellcook Biotechnology Co., Ltd (Guangzhou, China). 2,3,5-Triphenyltetrazolium chloride (TTC) was obtained from Solarbio (Beijing, China). ATP content chemiluminescence test kits, LDH colorimetric assay kits IL-10, IL-4, IL-1β, and TNF-α ELISA kits were purchased from Elabscience (Wuhan, China). ECAR and OCR fluorescence assay kits were purchased from Elabscience (Wuhan, China). The BCA protein assay kit was obtained from NCM Biotech (Suzhou, China), and the rProtein A/G magnetic IP/Co-IP kit was procured from ACE Biotech (Nanjing, China). ECL chemiluminescent substrate kits were acquired from Labgic (Beijing, China), with the Universal Two-step Kit and DAB Chromogenic Kit purchased from Zhongshan Jinqiao Biotechnology Co., Ltd (Beijing, China). Antibodies included CKLF1 (ab180512, Abcam,WB: 1:1,000; IHC/IF: 1:100; CoIP: 1:50, United Kingdom), CCR5 (ab11466, Abcam, IF: 1:80, United Kingdom), CCR5 (ab287959, Abcam, WB: 1:1,000, Uinted Kingdom), IL-1β (ab283822, Abcam, WB: 1:1,000, United Kingdom), anti-mTOR [Y391] (ab32028, Abcam, WB:1:1,000, United Kingdom), anti-mTOR (phospho S2481) (ab137133, Abcam, WB:1:1,000, UK), anti-AMPK alpha 1+AMPK alpha 2 (ab131512, Abcam,WB:1:1,000, United Kingdom), anti-AMPK alpha 1 (phospho T183)+AMPK alpha 2 (phospho T172) (ab133448, Abcam, WB:1:1,000, United Kingdom), anti-HIF-1α[EPR16897] (ab179483, Abcam, WB:1:1,000, United Kingdom), anti-β-actin (AC026, WB:1:10,000, ABclonal, United Kingdom), anti-PFKFB3 [EPR12594] (ab181861, Abcam, WB:1:1,000, United Kingdom), GLUT3 Polyclonal antibody (20403-1-AP, Wuhan Sanying, WB:1:1,000, China), HIF-1 alpha Antibody (AF1935-SP, Novus Biologicals, IF:1:100, United States),MAP2 Antibody (ab300645, Abcam, IF: 1:50, United Kingdom) β - Actin Antibody (AF7018, Wuhan Sanying, WB: 1:3,000, China), goat anti-mouse IgG H&L (HRP) (ab6789, Abcam, WB:1:1,000, United Kingdom)and goat anti-rabbit IgG (H + L) HRP (S0001, WB: 1:3,000) were obtained from Affinity Biosciences (Jiangsu, China). Cy3-labeled donkey anti-rabbit and FITC-labeled donkey anti-goat antibodies were purchased from Wuhan SafeTech Biotechnology Co. Mitochondrial Membrane Potential Assay Kit (JC-1) and One Step TUNEL Apoptosis Detection Kit were both purchased from Beyotime Biotechnology (China). Calcein AM/PI Double Staining Kit was acquired from Elabscience (United States), while Fluor 647-conjugated donkey anti-mouse IgG (H + L) was obtained from Life Technologies (California, United States).

### 2.2 Molecular docking and dynamics simulation

CKLF1 (UniProt: Q9UBR5-2) and NGR1 (PubChem:441,934) were converted to PDBQT and docked using AutoDock Vina. A docking box was built around the active site, conformations explored via genetic algorithm, and minimum binding energies ranked by multivariate regression. Results were visualized with PyMOL and Discovery Studio. Molecular dynamics (MD) simulations of the complex using GROMACS 2024.4 (Amber99sb-ildn/GAFF2) in a dodecahedral SPC/E water box (neutralized) included energy minimization (50,000 steps), NVT/NPT equilibration (100 ps each), and a 100 ns production run (2-fs timestep, 300 K, 1 bar). PME was used for electrostatics, and a 1.0-nm cutoff for van der Waals interactions (Van Der Spoel et al., 2005).

### 2.3 Cell thermal displacement assay

Cellular Thermal Shift Assay (CETSA) was performed using PC12 cell lysates. Lysates were treated with either vehicle (0.1% DMSO in PBS) or 10 μM NGR1 (37 °C, 1 h). Aliquots were then heated over a 40 °C–75 °C gradient (8 points, 3 min each) in a thermal cycler. Denatured proteins were pelleted by centrifugation (15,000 × g, 20 min, 4 °C), and soluble fractions were analyzed by Western blot using anti-CKLF1 antibody (with β-actin as loading control). Bands were quantified with ImageJ, and thermal stabilization curves were generated to determine half-maximal denaturation temperatures (Tm).

### 2.4 Drug affinity responsive target stability

The binding interaction between NGR1 and CKLF1 was evaluated using the DARTS assay. PC12 cell lysates were pretreated with 10 μM NGR1 or vehicle control (37 °C, 30 min), then digested with pronase (1:1,000 w/w) for 15 min at room temperature. Proteolysis was stopped by adding SDS-PAGE loading buffer and heating at 95 °C for 10 min. After centrifugation, total protein in supernatants was quantified by BCA assay. Equal amounts (80 μg/lane) were resolved by SDS-PAGE, transferred to PVDF membranes, and immunoblotted using anti-CKLF1 antibody (with β-actin as loading control).

### 2.5 PC12 cell culture and oxygen glucose deprivation model construction

PC12 cell line with high differentiation status was obtained from Guangzhou Cellcook Biotechnology Co., Ltd (Guangzhou, China). Cells were thawed and cultured in RPMI-1640 medium supplemented with 10% fetal bovine serum and 1% penicillin/streptomycin at 37 °C under 5% CO_2_. Upon reaching 80% confluency, cells were pretreated for 24 h with graded concentrations of NGR1 diluted in serum-free medium, based on prior dose-response studies (Zhou et al., 2019; Li et al., 2022). To induce ischemic-like conditions, cultures were transitioned to glucose-free medium and placed in a hypoxia chamber. The chamber was flushed with 95% N_2_/5% CO_2_ until oxygen levels, monitored via a calibrated oximeter, stabilized below 0.5%. Inlet/outlet valves were then sealed to maintain anoxia. This OGD protocol was maintained for 6 h to recapitulate cerebral ischemia pathophysiology.

#### 2.6 CCK-8 assay for cytotoxicity and screening of optimal dosing concentration

Cell viability was quantified using the Cell Counting Kit-8 (CCK-8) colorimetric assay. PC12 cells were seeded into 96-well plates at a density of 1 × 10^4^ cells/well (100 μL/well) and allowed to adhere for 24 h in a humidified 5% CO_2_ incubator at 37 °C. Cells were pretreated with graded concentrations of NGR1 (0.01–100 μM) for 24 h prior to OGD induction. Following OGD, 10 μL of CCK-8 reagent was added to each well, and plates were incubated for 2 h under standard culture conditions. Absorbance was measured at 450 nm using a microplate reader, with reference wavelength set at 650 nm. Cell viability was normalized to vehicle-treated control groups (0.1% DMSO) and expressed as a percentage of viable cells. Dose-response curves were generated to determine the optimal therapeutic concentration of NGR1.

### 2.7 PC12 cell grouping

After cell concentration detection and administration concentration screening, the experimental groupings were determined: (1) blank control group (Control), (2) solvent control group (DMSO), (3) model group (OGD), (4) NGR1 low-dose group (0.1 μM), (5) NGR1 medium-dose group (1 μM), and (6) NGR1 high-dose group (10 μM), and various kinds of drugs were administered and molded, and then followed by subsequent experimental assays.

### 2.8 Live-dead staining

PC12 cells were inoculated into 24-well plates for drug administration and modelling; the working solution was configured according to the kit instructions; 200 μL of staining working solution was added to each well; and the cells were incubated at 37 °C for 10–30 min, and the staining effect was observed under a Primovert fluorescence microscope (Zeiss, Germany) at the end of incubation.

### 2.9 TUNEL detection of PC12 cell apoptosis

PC12 cells were inoculated into 24-well plates containing slides for drug administration and modeling, followed by the addition of an appropriate amount of fixative to fix the cells, washed with PBS, and then added with PBS containing 0.3% Trinton X-100 for permeabilization for 5 min. The assay solution was configured according to the kit instructions, 50 μL of TUNEL assay solution was added to each well, and the cells were incubated for 60 min at 37 °C, protected from light, and subsequently PBS was washed twice, and DAPI containing anti-fluorescence bursting agent was added to seal the film, which was placed under the fluorescence microscope for observation.

### 2.10 JC-1 detection of mitochondrial membrane potential changes

PC12 cells were inoculated into 6-well plates for administration and modelling, staining working solution and buffer were configured according to the kit instructions. 1 mL of JC-1 staining working solution was added to each well, incubated at 37 °C for 20 min, followed by washing with buffer, and finally 2 mL of cell culture solution was added for observation under a Primovert fluorescence microscope (Zeiss, Germany).

### 2.11 PC12 cell metabolism assay

The metabolic tests are lactate dehydrogenase, lactate, ATP, ECAR,and OCR. LDH, lactate, ATP release, and ECAR and OCR changes in PC12 cells after OGD were detected using kits and a Varioskan LUX enzyme labeller (Thermo Fisher Scientific, United States), respectively.

### 2.12 Animals

Wild-type (WT) SD rats were sourced from Slaughter Jingda Laboratory Animal Co., Ltd (Hunan, China), while CKLF1-knockout (CKLF1−/−) rats were obtained from the Institute of Pharmaceutical Research at the Chinese Academy of Medical Sciences (Beijing, China). All animal procedures were approved by and conducted in compliance with the guidelines of the Animal Ethics Committee of Hunan University of Traditional Chinese Medicine.

### 2.13 Construction of the permanent middle cerebral artery occlusion (pMCAO) model and drug grouping

WT Group. One hundred and twenty WT male Sprague-Dawley (SD) rats (250–280 g) were randomly allocated via simple random sampling into six groups with twenty rats per group, namely: (1) Sham group; (2) Model group; (3) NGR1 10 mg/kg group; (4) NGR1 20 mg/kg group; (5) NGR1 40 mg/kg group; (6) Edaravone group.

CKLF1−/− Group. For the CKLF1 gene knockout experiment, sixty WT male SD rats (250–280 g) and another sixty CKLF1−/− male SD rats (250–280 g) were each randomly allocated via simple random sampling into three groups with fifteen rats per group, yielding six experimental groups in total: (1) WT Sham group; (2) WT Model group; (3) WT NGR1 40 mg/kg group; (4) CKLF1−/− Sham group; (5) CKLF1−/− Model group; (6) CKLF1−/− NGR1 40 mg/kg group.

Rats were anesthetized with 1%–2% isoflurane carried by a mixture of 30% O_2_ and 70% N_2_O. The common carotid artery (CCA), external carotid artery (ECA) and internal carotid artery (ICA) were surgically isolated. The distal CCA bifurcation was ligated, and the ICA was temporarily occluded with a microvascular clip. For the pMCAO group, a nylon filament was inserted into the ICA to permanently block the middle cerebral artery. Sham-operated rats underwent identical surgical manipulations without induction of cerebral ischemia. All rats recovered on a thermostatically controlled electric blanket with *ad libitum* access to food and water postoperatively.

pMCAO rats received intraperitoneal test drug at 6 h post-ischemia, with sample processing performed 3 h later (9 h after ischemia onset). Sham rats were injected intraperitoneally with an equal volume of sterile normal saline at 6 h post-sham surgery, and samples were collected at the matched 9 h time point.

### 2.14 TTC staining and neurofunctional behavioral tests

Eight rats from each group underwent TTC staining and neurobehavioral testing to evaluate cerebral infarction and neurological deficits. Following confirmed anesthesia, whole brains were carefully removed and sectioned into six uniform coronal slices. These sections were stained with 1.5% TTC in phosphate-buffered saline (PBS) at 37 °C for 15 min, then flipped and stained for another 15 min in the dark. Infarct and edema rates were quantified using GraphPad Prism 9.0, calculated as: infarct rate (%) = (total infarct area/total cross-sectional area) × 100%, and edema rate (%) = (left hemisphere area/right hemisphere area) × 100%. Neurobehavioral assessments included the Zea Longa test (0–5 points), forelimb slip test, suspension test, and mesh screen test to determine functional nervous system impairment. (Long et al., 2024). All behavioral assessments were conducted by investigators blinded to group assignments, and the data were analyzed using GraphPad Prism 9.0 software.

### 2.15 Nissl staining

Neuronal damage was assessed by Nissl staining with toluidine blue. Anesthetized rats were perfused with 4% paraformaldehyde, and brains were post-fixed in 4% formalin. Paraffin-embedded sections were deparaffinized, stained with toluidine blue, cleared in xylene (10 min), mounted with neutral resin, and imaged using a digital slide scanner (3DHISTECH, Hungary).

### 2.16 Enzyme-linked immunosorbent assay (ELISA)

ELISA was used to detect the expression levels of IL-1β, TNF-α, IL-10, and IL-4 in the serum of each group of rats. The expression levels of the above proteins were assessed using ELISA kits, all experimental steps were performed by the same laboratory staff, and the final results were analyzed using GraphPad Prism 9.0 software.

### 2.17 Immunohistochemical staining (IHC)

Tissue sections were processed, deparaffinized, treated with 3% H_2_O_2_ (10 min, RT) to block endogenous peroxidase, and blocked with 5% BSA (1 h, 37 °C). Sections were incubated overnight at 4 °C with primary antibody, followed by secondary antibody (1 h, 37 °C). DAB substrate was used for chromogenic development; the reaction was stopped when a brown precipitate appeared. Sections were counterstained with hematoxylin, dehydrated, and coverslipped. Whole-slide images were acquired using a digital scanner, analyzed with ImageJ, and quantified from six random fields per group using GraphPad Prism 9.0.

### 2.18 Immunofluorescence colocalization (IF)

CKLF1 expression and co-localization with CCR5/HIF-1α were assessed by IF in PC12 cells and brain sections. Deparaffinized sections were treated with 3% H_2_O_2_ (10 min, RT), blocked with 5% BSA (1 h, 37 °C), and incubated with primary antibody overnight at 4 °C, followed by fluorescent secondary antibody (1 h, 37 °C, dark). Sections were mounted with DAPI-containing anti-fade medium. Images were captured using a confocal microscope, and six random fields per group were analyzed with ImageJ and GraphPad Prism 9.0.

### 2.19 Western blot assay (WB)

Treated PC12 cells or brain tissues (hippocampus and cortex) were selected for protein extraction, and the extracted proteins were subjected to BCA quantification to prepare WB samples, followed by electrophoresis, membrane transfer and closure, and the closed PVDF membranes were incubated with primary antibodies (CKLF1, CCR5, IL-1β, AMPK, p-AMPK, mTOR, p-mTOR, HIF-1α, PFKFB3, GLUT3) overnight at 4 °C. To block non-specific binding, HRP-labelled affinity-purified sheep anti-rabbit IgG (H + L) was used as the secondary antibody and incubated at room temperature for 1 h. Protein expression levels were detected by an ultra-sensitive chemiluminescence imager (Amersham Imager 600, General Electric Company, United States). The grayscale values of protein bands were quantified using ImageJ, and all data were subsequently analyzed with GraphPad Prism 9.0 software.

### 2.20 Co-immunoprecipitate assay (Co-IP)

Protein A agarose beads were incubated with the samples overnight at 4 °C. The following day, the beads were washed three times with wash buffer and centrifuged at 3,000 rpm for 5 min at 4 °C. Antibodies against CKLF1, CCR5, and HIF-1α were then added to precipitate the target proteins. Subsequent detection was carried out according to standard WB procedure. To control for non-specific binding, IgG and Input controls were included. Protein expression was detected using an HRP-conjugated goat anti-rabbit IgG (H + L) secondary antibody, followed by imaging with an ultrasensitive chemiluminescent system. Band intensities were quantified in ImageJ, and statistical analysis was performed with GraphPad Prism 9.0.

### 2.20 Statistical analyses

Statistical analysis was conducted in GraphPad Prism 9.0, which was used to assess data normality and identify outliers. The data were normalized relative to the control group or sham-operated group, whose values were set to 1. Results are expressed as mean ± standard deviation (SD). Differences among groups were evaluated by one-way ANOVA, followed by Tukey’s *post hoc* test for multiple comparisons. A threshold of P < 0.05 was considered statistically significant. The sample size for each experiment is indicated in the corresponding figure legends.

## 3 Results

### 3.1 NGR1 is protective after PC12 oxygen glucose deprivation

To investigate the neuroprotective potential of NGR1, we utilized an *in vitro* oxygen-glucose deprivation (OGD) model in PC12 cells (Figure 1A). Initial cytotoxicity screening via the CCK-8 assay revealed no adverse effects of NGR1 at concentrations ranging from 0.1 to 100 μM (Figures 1B,C). Subsequent OGD challenges demonstrated maximal neuroprotection at 10 μM NGR1, with no significant enhancement at a higher dose (100 μM). Based on these findings, further experiments employed NGR1 at 0.1, 1, and 10 μM to validate its dose-dependent efficacy. Live/dead staining confirmed that OGD induced widespread neuronal death, whereas NGR1 treatment significantly reduced cell mortality in a concentration-dependent manner (Figures 1E,F). These results underscore NGR1’s capacity to preserve neuronal viability under ischemic conditions, highlighting its therapeutic potential for cerebral ischemia.

**FIGURE 1:**
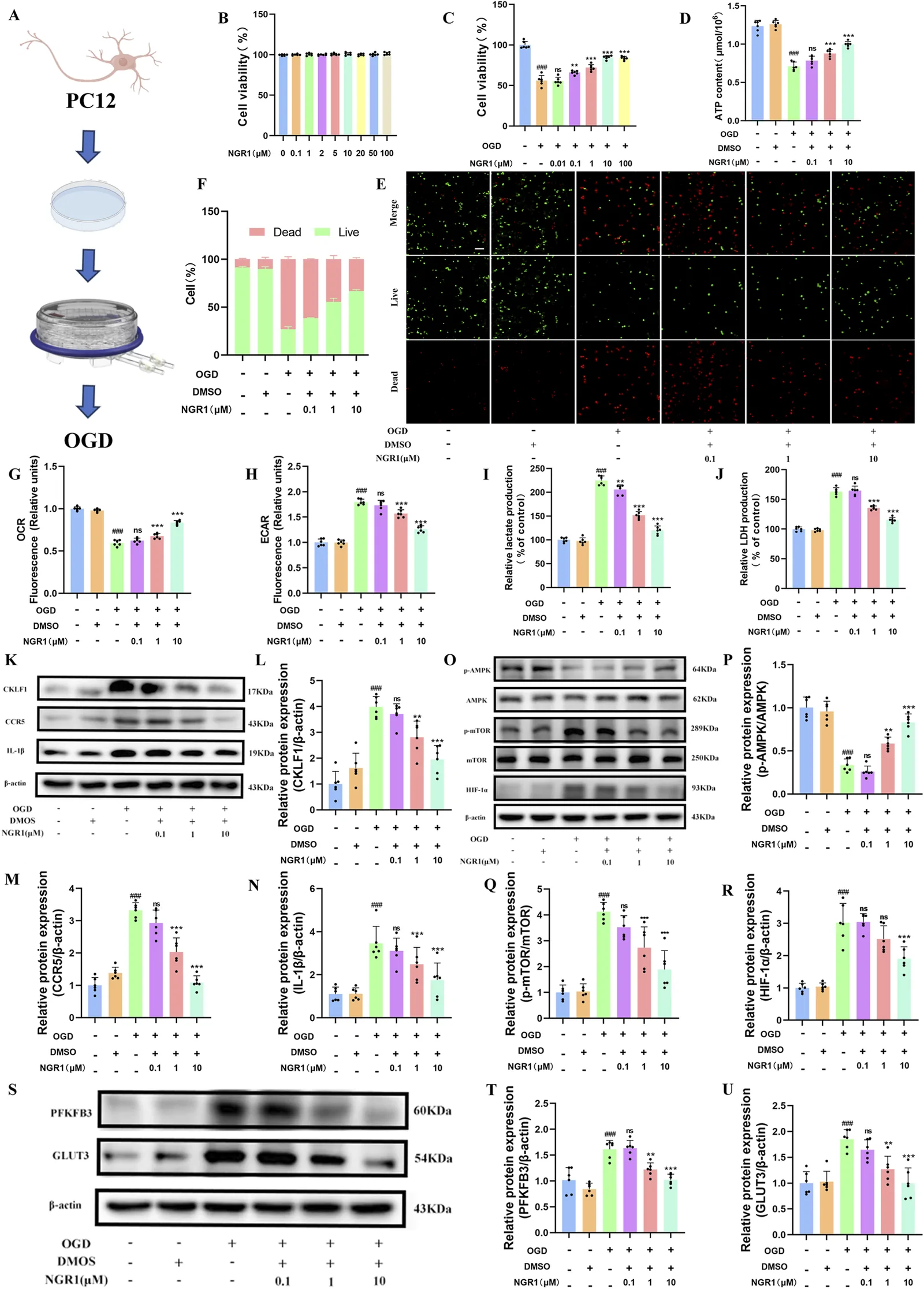
NGR1 has a protective effect on neuronal cells after OGD, depending on the CKLF1/CCR5 axis to regulate AMPK/mTOR/HIF-1α-mediated metabolic reprogramming. **(A)** The flowchart of simulating the pathological state of ischemic stroke by oxygen glucose deprivation using PC12 cells. **(B)** The cytotoxicity assay. **(C)** The effect of NGR1 on cell viability after OGD in PC12 cells. **(D)** The level of ATP production. **(E,F)** The graphs of the results of live-dead staining. **(G)** The relative level of cellular OCR. **(H)** The relative level of cellular ECAR. **(I)** The relative level of cellular lactate production. **(J)** The relative level of cellular LDH release. **(K**‑**N)** The results and analysis of WB determination of cellular CKLF1/CCR5 axis and IL-1β protein expression levels. **(O**‑**R)** The results and analysis of WB determination of cellular AMPK/mTOR/HIF-1α signaling pathway expression levels. **(S**‑**U)** The results and analysis of WB determination of PFKFB3 and GLUT3 protein expression levels. Magnification ×40, scale bars, 100 μm. All data results were expressed as mean ± SD (n = 6). ^#^ *p* < 0.05 vs. control; ^##^ *p* < 0.01 vs. control; ^###^ *p* < 0.001 vs. control; ^*^ *p* < 0.05 vs. model; ^**^ *p* < 0.01 vs. model; ^***^ *p* < 0.001 vs. model.

To investigate metabolic perturbations in neurons following ischemic injury, we assessed ATP production, mitochondrial function, and cytotoxic markers in PC12 cells subjected to OGD. Chemiluminescence assays revealed a significant reduction in intracellular ATP levels after OGD, which were restored by treatment with 10 μM NGR1 (Figure 1D). Metabolic flux analysis demonstrated that OGD impaired mitochondrial oxidative phosphorylation (as evidenced by a reduced oxygen consumption rate, OCR) and enhanced glycolytic activity (as indicated by an elevated extracellular acidification rate, ECAR). NGR1 intervention reversed these alterations, normalizing OCR and ECAR to near-baseline levels (Figures 1G,H). Cytotoxicity assays further indicated that OGD exacerbated LDH release and lactate accumulation, both hallmarks of ischemic injury. NGR1 treatment significantly attenuated both LDH leakage and lactate overproduction, underscoring its capacity to mitigate metabolic and cytotoxic stress (Figures 1I,J). Collectively, these findings demonstrate that NGR1 corrects ischemia-driven metabolic dysfunction by restoring bioenergetic equilibrium and reducing glycolytic flux, positioning it as a promising therapeutic agent for cerebral ischemia.

To elucidate the mechanisms underlying neuronal metabolic reprogramming following ischemia, we investigated the CKLF1/CCR5 axis and associated signaling pathways in PC12 cells subjected to OGD. Western blot analysis revealed that OGD significantly upregulated the pro-inflammatory mediators CKLF1, CCR5, and IL-1β, while treatment with 10 μM NGR1 attenuated these increases (Figures 1K–N). OGD suppressed AMPK phosphorylation while enhancing mTOR phosphorylation and HIF-1α expression, indicative of disrupted metabolic homeostasis. NGR1 reversed these effects, restoring AMPK activity and suppressing mTOR/HIF-1α signaling (Figures 1O–R). Concomitantly, OGD elevated the glycolytic rate-limiting enzymes PFKFB3 and GLUT3, and NGR1 normalized their expression (Figures 1S–U).

Increased lactate production paralleled these glycolytic shifts, corroborating metabolic reprogramming toward anaerobic glycolysis. IF analysis confirmed that in OGD-treated cells, the fluorescence intensity of CKLF1, CCR5 and HIF-1α proteins significantly increased, with markedly enhanced colocalization. Conversely, NGR1 significantly reduced the fluorescence intensity of these proteins and diminished this colocalization phenomenon. (Figures 2A–D). This suggests that CKLF1/CCR5 signaling drives HIF-1α-mediated glycolytic adaptation under ischemia. TUNEL staining demonstrated that OGD-induced apoptosis was mitigated by NGR1 (Figures 2E,F). JC-1 fluorescence revealed a collapse in mitochondrial membrane potential (ΔΨm) post-OGD, characterized by a red-to-green fluorescence shift (Perelman et al., 2012). NGR1 preserved ΔΨm, indicating restored mitochondrial function (Figures 2G,H). NGR1 attenuates ischemic neuronal injury by suppressing the CKLF1/CCR5 axis, thereby rectifying AMPK/mTOR/HIF-1α dysregulation, normalizing glycolytic flux, and preserving mitochondrial integrity. These findings position NGR1 as a therapeutic agent capable of counteracting metabolic reprogramming and apoptosis in cerebral ischemia.

**FIGURE 2:**
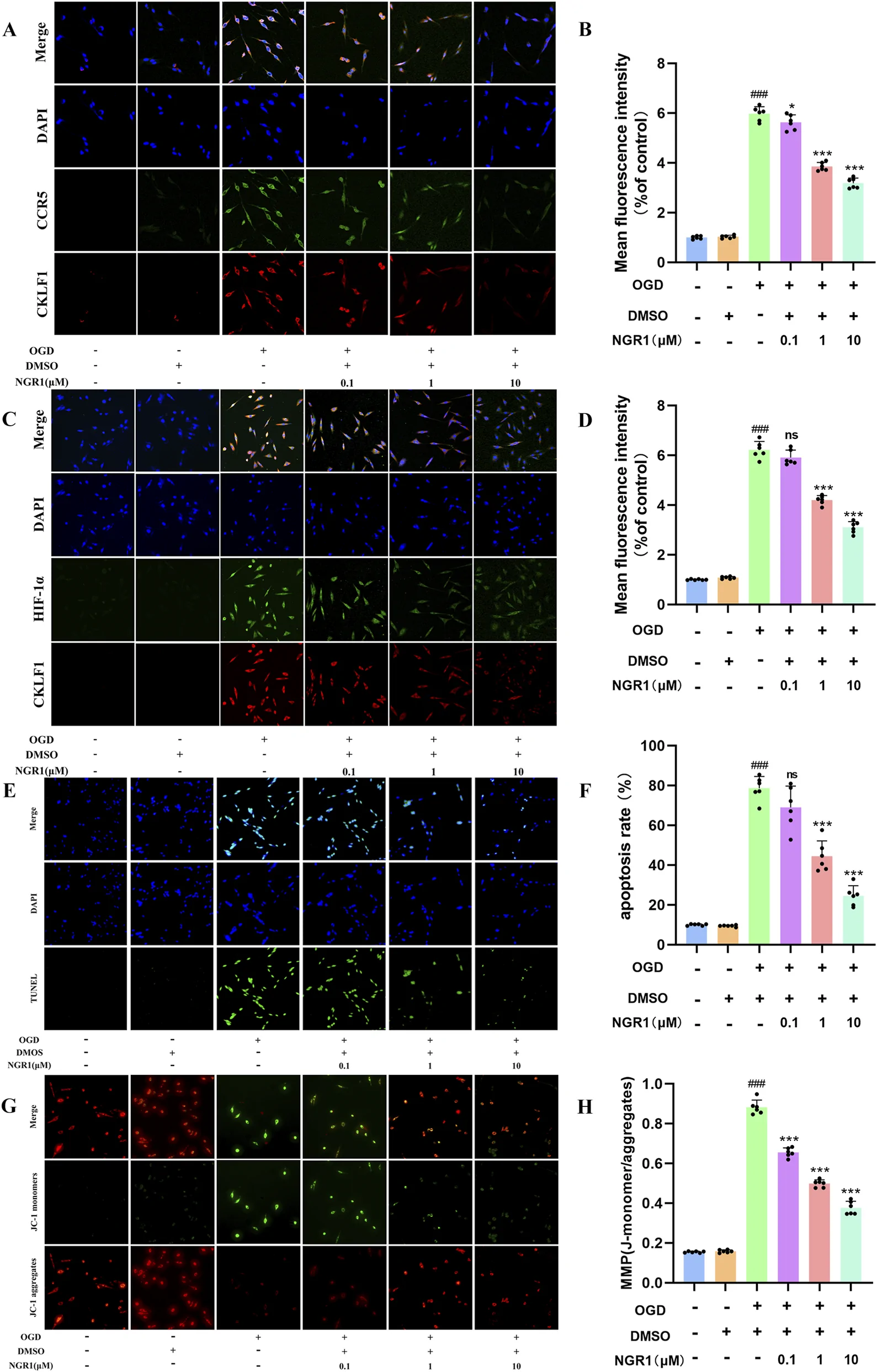
Fluorescence co-localization of CKLF1/CCR5 and CKLF1/HIF-1α after inhibition of neuronal OGD by NGR1, and inhibits neuronal apoptosis. **(A**‑**D)** The results and analysis of fluorescence co-localization of cellular CKLF1/CCR5 and CKLF1/HIF-1α. **(E,F)** TUNEL staining results and analysis. **(G,H)** JC-1 staining results and analysis (n = 6). Magnification ×40, scale bars, 100 μm. All data results were expressed as mean ± SD. ^#^ *p* < 0.05 vs. control; ^##^ *p* < 0.01 vs. control; ^###^ *p* < 0.001 vs. control; ^*^ *p* < 0.05 vs. model; ^**^ *p* < 0.01 vs. model; ^***^ *p* < 0.001 vs. model.

### 3.2 CKLF1 is a binding target protein of NGR1

Molecular docking simulations predicted a robust binding affinity between NGR1 and CKLF1, with a calculated binding energy of −7.4 kcal/mol, and revealed hydrogen bonding interactions with CKLF1 residues Lys9, Asp27, Asn30, and Thr34. Structural analysis revealed hydrogen-bonding interactions at multiple residues (Figure 3A), suggesting that CKLF1 is a direct pharmacological target of NGR1. All-atom molecular dynamics simulations (100 ns) performed using GROMACS elucidated the structural dynamics and binding stability of the NGR1–CKLF1 complex. Trajectory analysis quantified five key parameters: root-mean-square deviation (RMSD), root-mean-square fluctuation (RMSF), hydrogen-bond number, radius of gyration (Rg), solvent-accessible surface area (SASA) (Figures 3B–F), and MM-GBSA binding free energy (ΔGMMGBSA). The complex achieved dynamic equilibrium across the simulation trajectory, with RMSD values fluctuating within a narrow range despite a slight upward drift at the late simulation stage. RMSF analysis identified moderate flexibility within structured core domains, while prominent flexibility was observed at the N-terminal and C-terminal segments, especially the disordered C-terminal tail. Persistent dynamic hydrogen-bond interactions were maintained throughout the simulation. Consistently compact conformation was demonstrated by limited Rg oscillations, and SASA underwent initial conformational compaction followed by steady-state oscillation. Thermodynamic analysis revealed favorable binding affinity (ΔGMMGBSA = −69.345 kcal/mol), further corroborating the stable binding behavior of the NGR1-CKLF1 complex. Collectively, these metrics confirm that the NGR1-CKLF1 complex maintains structural integrity, dynamic equilibrium, and predicted stable binding interaction under physiological conditions.

**FIGURE 3:**
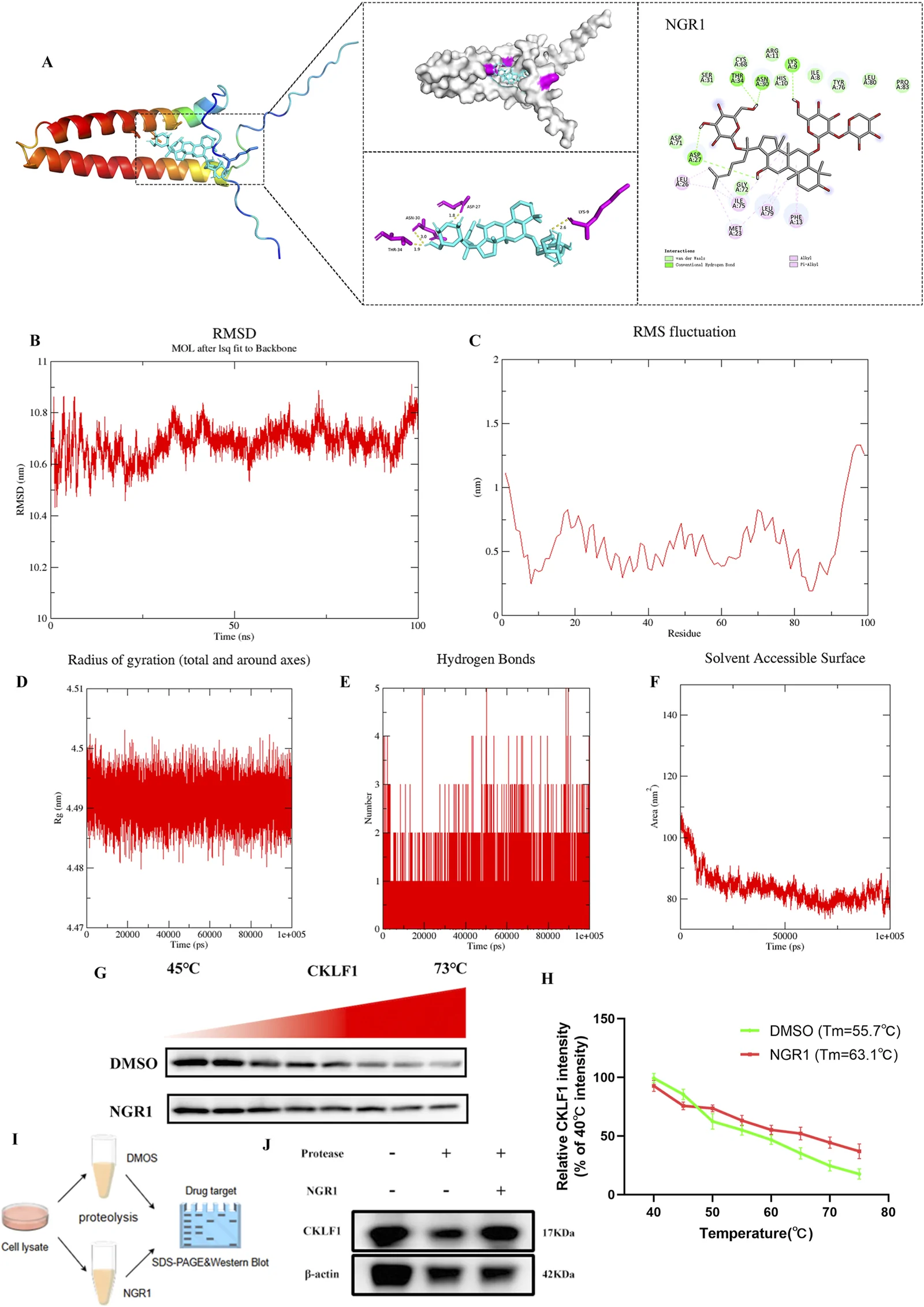
CKLF1 stably interacts with NGR1 to form a complex, where CKLF1 is the target of NGR1. **(A)** The results of molecular docking of NGR1 with CKLF1. **(B**‑**F)** The root-mean-square deviation (RMSD), root-mean-square fluctuation (RMSF), radius of gyration (Rg), hydrogen bonds and solvent accessible surface area (SASA) of the complex of CKLF1 with NGR1. **(G,H)** The CETSA results and analysis. **(I,J)** The schematic diagram and results of the DARTS technique (n = 3).

We carried out CETSA to experimentally validate this protein interaction. Thermal denaturation curves showed that NGR1 markedly enhanced CKLF1 thermal stability, causing an obvious right shift in Tm by +7.4 °C relative to vehicle controls (Figures 2G,H). This stabilization indicates direct ligand–target binding. DARTS assays further corroborated these findings. Co-incubation of PC12 cell lysates with NGR1 and promise reduced the proteolytic degradation of CKLF1, whereas β-actin levels remained unchanged, confirming selective stabilization (Figures 3I,J). Together, these data establish that NGR1 directly binds to and stabilizes CKLF1 through specific molecular interactions, positioning CKLF1 as a critical mediator of the anti-ischemic effects of NGR1.

### 3.3 Protective effects of NGR1 against permanent focal cerebral ischemic injury

We first assessed the *in vivo* neuroprotective efficacy of NGR1 via TTC staining and multiple neurobehavioral assays. Compared with the sham group, pMCAO operation markedly elevated cerebral infarct and edema volumes (Figures 4A–C). Intervention with graded NGR1 or positive control Edaravone significantly alleviated such pathological swelling and infarction. Consistently, severe neurological dysfunction was observed in model rats, whereas both agents remarkably rescued behavioral deficits (Figures 4D–G). Collectively, NGR1 mitigates permanent cerebral ischemic damage by reducing infarct/edema severity and restoring neurological function.

**FIGURE 4:**
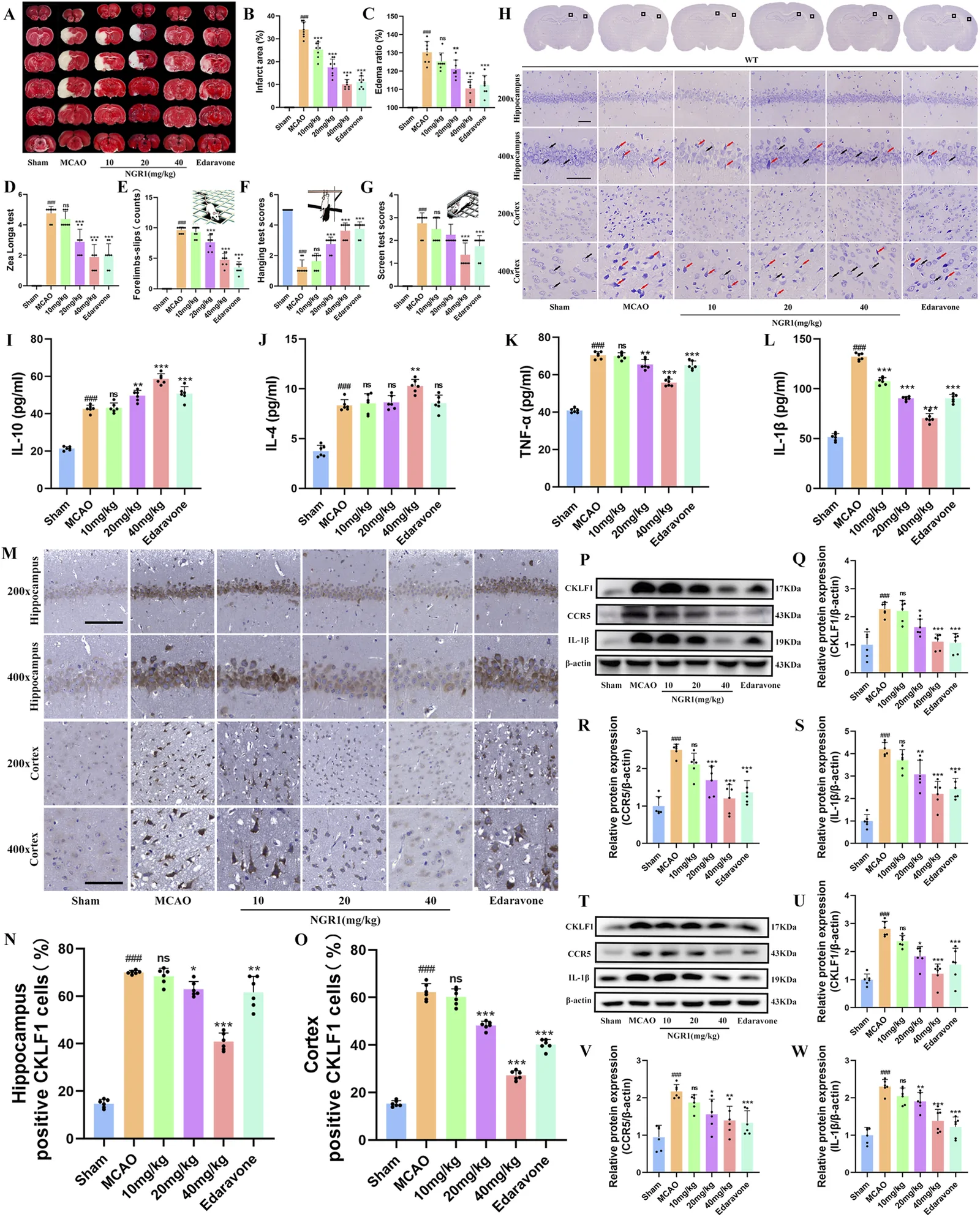
Pharmacodynamic testing of NGR1 against ischemic stroke and its ability to inhibit the CKLF1/CCR5 axis after cerebral ischemia and improve the inflammatory response after cerebral ischemia. **(A**‑**C)** The TTC staining and quantitative analysis of cerebral infarction and edema rates. **(D**‑**G)** The results of neurobehavioral tests, including the Zea Longa score, forelimb slip test, screen test, and hanging test (n = 8). **(H)** The Nissl staining of the hippocampal CA1 region and cortex (red arrows: damaged neurons; black arrows: normal neurons). **(I**‑**L)** The serum cytokine levels (IL-10, IL-4, TNF-α, IL-1β) measured by ELISA. **(M**‑**O)** The IHC staining and quantification of CKLF1 protein. **(P**‑**W)** represent WB analysis of the CKLF1/CCR5 axis and IL-1β expression in hippocampal and cortical tissues (n = 6). All quantitative data are presented as mean ± SD. Scale bars: 50 μm (200×) and 20 μm (400×). ^#^ *p* < 0.05 vs. sham; ^##^ *p* < 0.01 vs. sham; ^###^ *p* < 0.001 vs. sham; ^*^ *p* < 0.05 vs. model; ^**^ *p* < 0.01 vs. model; ^***^ *p* < 0.001 vs. model.

Nissl staining further visualized neuronal morphological lesions in the cortex and hippocampal CA1 region (Figure 4H). Sham rats exhibited intact, plump, densely packed neurons with clear Nissl bodies. In contrast, model rats displayed shrunken, sparsely distributed neurons with condensed staining. NGR1 and Edaravone both reversed neuronal morphological deterioration, verifying their neuroprotective activity.

ELISA quantification of serum inflammatory cytokines revealed drastically increased pro-inflammatory IL-1β, TNF-α and elevated anti-inflammatory IL-4, IL-10 after pMCAO (Figures 4I–L). NGR1 and Edaravone simultaneously suppressed pro-inflammatory mediators and upregulated anti-inflammatory cytokines, indicating robust anti-inflammatory capacity against post-ischemic neuroinflammation.

### 3.4 NGR1 represses CKLF1-mediated neuronal metabolic reprogramming post cerebral ischemia

IHC was performed to detect CKLF1 protein abundance in rat cortex and hippocampus to clarify whether NGR1 targets the CKLF1 cascade *in vivo*. pMCAO induced prominent CKLF1 overexpression, which was dose-dependently suppressed by 10–40 mg/kg NGR1 (Figures 4M–O). Moreover, NGR1 exerted stronger inhibitory effects on CKLF1 immunoreactivity than the equal-dose Edaravone control. WB further validated this regulatory pattern: NGR1 intervention significantly blocked the activation of the CKLF1/CCR5 axis and downstream IL-1β overproduction (Figures 4P–W).

To further elucidate the downstream signaling cascade regulated by the CKLF1/CCR5 axis underlying NGR1’s anti-ischemic mechanism, WB was used to detect key proteins in the hippocampus and cortex of pMCAO rats. NGR1 markedly restored the disrupted AMPK/mTOR/HIF-1α signaling cascade (Figures 5A–H). Consistently, NGR1 downregulated the glycolytic mediators PFKFB3 and GLUT3, recapitulating our *in vitro* cellular observations (Figures 5I–N). The IF co-staining further revealed robust co-localization of CKLF1 with CCR5 and HIF-1α in MAP2^+^ neurons after cerebral ischemia; this protein interaction was attenuated by NGR1 in a dose-dependent manner (Figures 5O–V). Although Edaravone exerted comparable regulatory effects, its potency was inferior to NGR1 in disrupting CKLF1-CCR5 and CKLF1-HIF-1α complexes.

**FIGURE 5:**
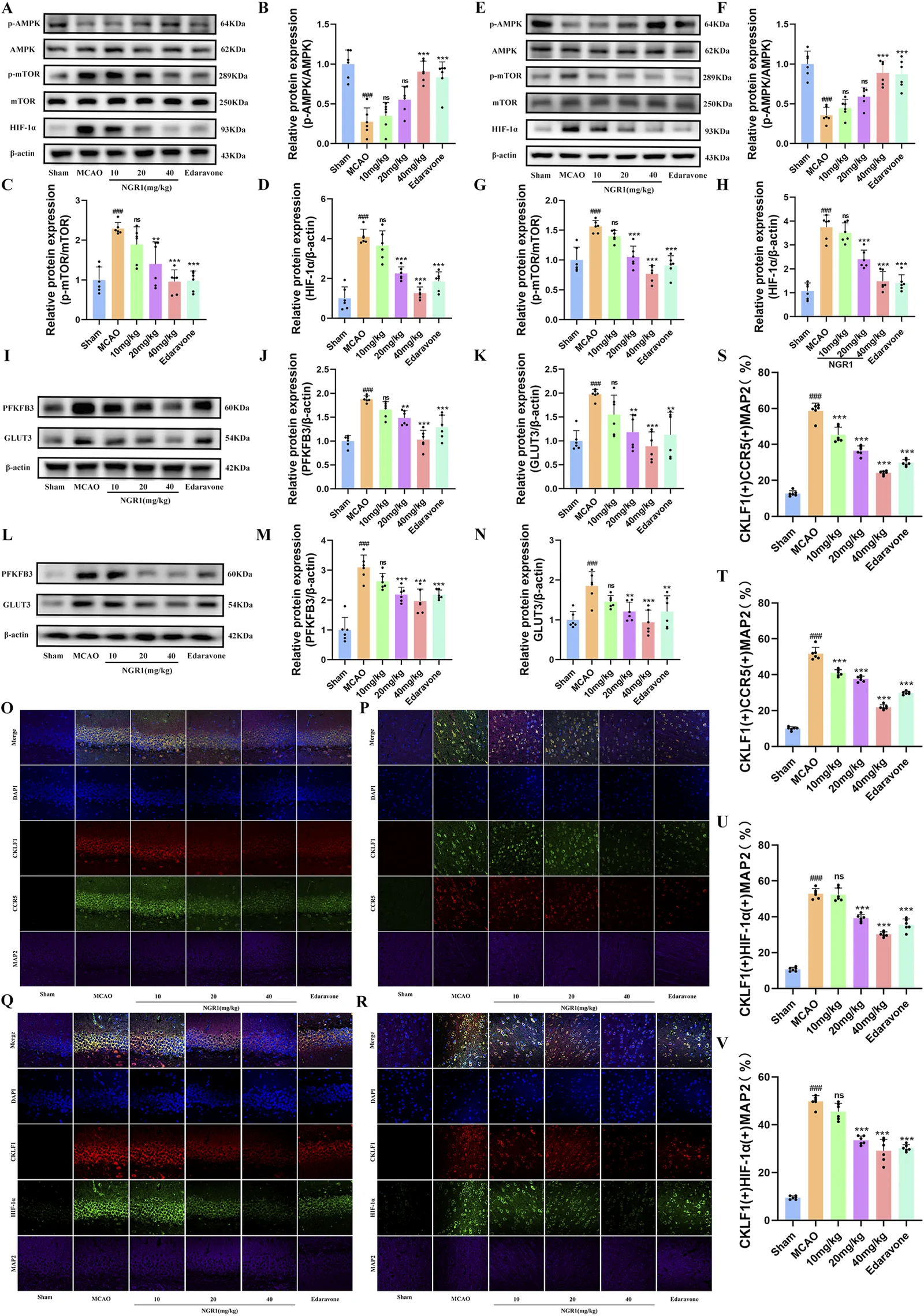
NGR1 modulates neuronal metabolic reprogramming by regulating CKLF1, which acts through the AMPK/mTOR/HIF-1α signaling pathway, thereby attenuating neuronal glycolytic activity. **(A**‑**H)** The results and analyses of WB determination of hippocampal and cortical AMPK/mTOR/HIF-1α signaling pathway expression. **(I**‑**N)** The results and analysis of WB determination of PFKFB3 and GLUT3 protein expression in the cortex and hippocampus. **(O**‑**V)** The results and analysis of fluorescence co-localization of CKLF1/CCR5/MAP2 and CKLF1/HIF-1α/MAP2 in the hippocampus and cortex. Magnification ×40, scale bars, 100 μm. All data results were expressed as mean ± SD (n = 6). ^#^ *p* < 0.05 vs. sham; ^##^ *p* < 0.01 vs. sham; ^###^ *p* < 0.001 vs. sham; ^*^ *p* < 0.05 vs. model; ^**^ *p* < 0.01 vs. model; ^***^ *p* < 0.001 vs. model.

Co-IP assays verified strong binding between CKLF1 and CCR5, as well as CKLF1 and HIF-1α in the hippocampus and cortex of pMCAO rats (Figures 6A–Q). Treatment with 40 mg/kg NGR1 substantially weakened these pathogenic protein-protein complexes.

**FIGURE 6:**
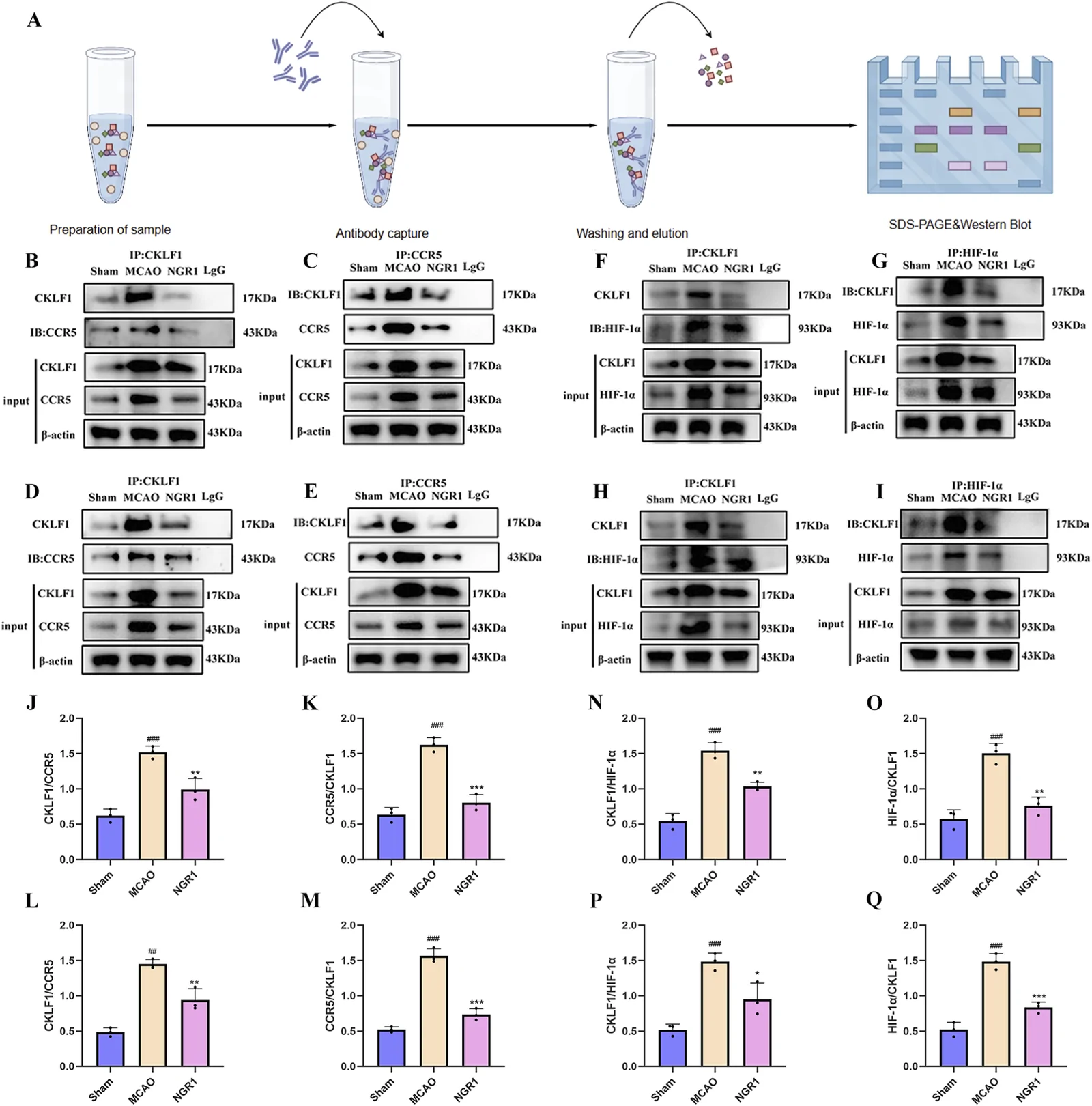
Immunoprecipitation of CKLF1 with CCR5 and CKLF1 with HIF-1α after cerebral ischemia, and NGR1 can inhibit its binding effect. **(A)** The flowchart of the Co-IP experiment. **(B**‑**E,J**‑**M)** The results and analyses of co-precipitation of CKLF1 with CCR5 and co-precipitation of CKLF1 with CCR5 in the cortex and hippocampus. **(F**‑**I,N**‑**Q)** The results and analyses of co-precipitation of CKLF1 with HIF-1α and co-precipitation of CKLF1 with HIF-1α in the cortex and hippocampus (n = 3). All data results were expressed as mean ± SD. ^##^ *p* < 0.01 vs. sham; ^###^ *p* < 0.001 vs. sham; ^*^ *p* < 0.05 vs. model; ^**^ *p* < 0.01 vs. model; ^***^ *p* < 0.001 vs. model.

Collectively, NGR1 exerts dual protective effects against cerebral ischemia: it blocks CKLF1/CCR5 signaling to restrain neuroinflammation, and rescues disordered AMPK/mTOR/HIF-1α cascades to normalize glycolytic metabolism. Restored bioenergetics further reduces neuronal apoptosis. Compared with Edaravone, NGR1 exhibits superior efficacy in dissociating harmful CKLF1-containing complexes and correcting ischemic metabolic dysfunction, highlighting its translational potential for ischemic stroke treatment.

### 3.5 CKLF1 knockdown results in a significant reduction of NGR1 in the treatment of ischemic stroke

To further verify CKLF1 as the therapeutic target of NGR1 in ischemic stroke, CKLF1−/− knockout rats were utilized. TTC staining showed that after cerebral ischemia, CKLF1−/− rats displayed smaller infarct volumes, alleviated cerebral edema and improved neurological scores relative to wild-type (WT) rats (Figures 7A–D). Notably, NGR1 treatment failed to produce additional protective effects on brain edema, infarct injury or neurological function in CKLF1−/− rats.

**FIGURE 7:**
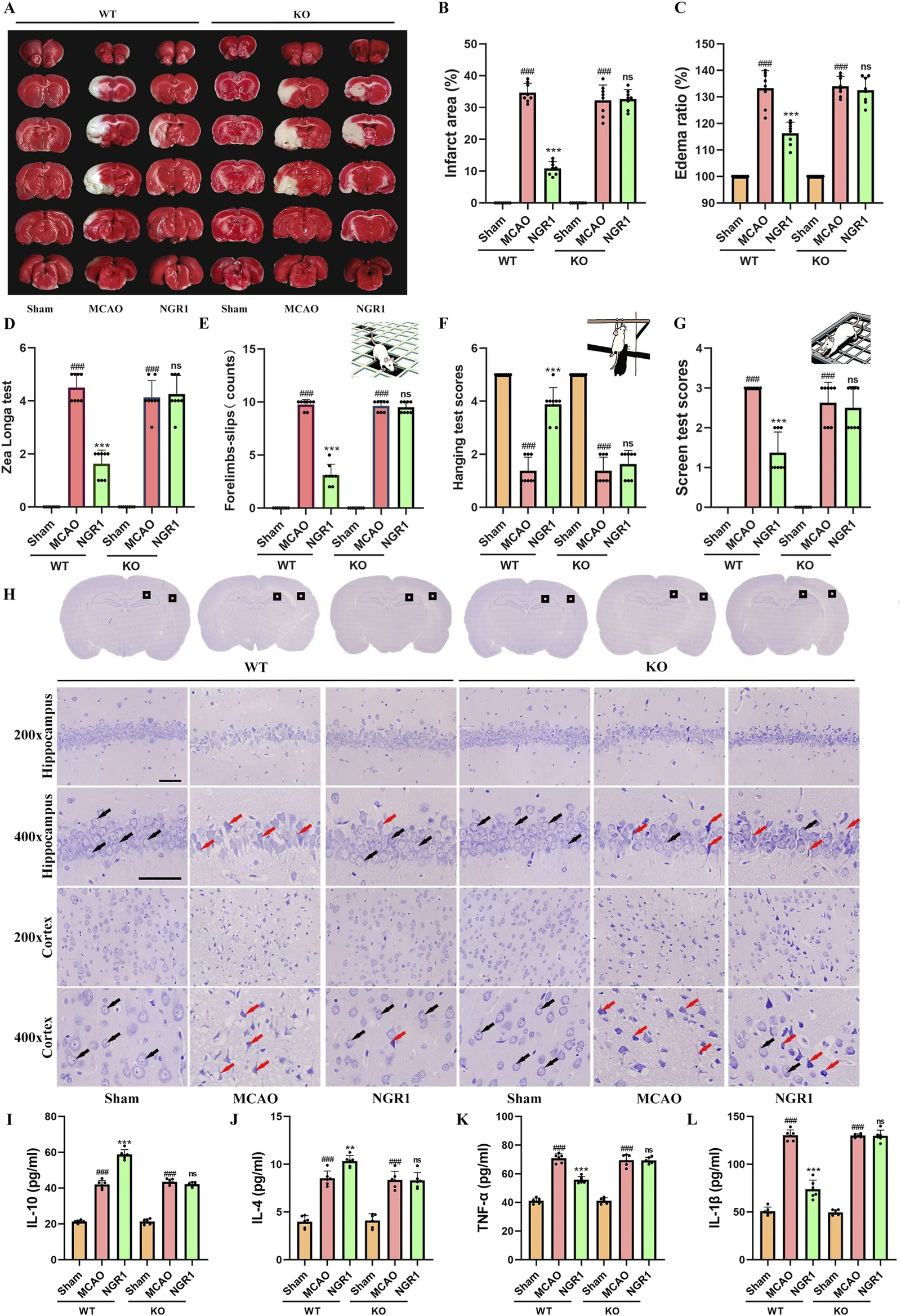
Knockdown of the CKLF1 gene deprived NGR1 of its therapeutic effect on ischemic stroke. **(A**‑**C)** The TTC-stained brain sections and the corresponding quantitative analysis of cerebral infarction and edema rates after 9 h of ischemia. **(D**‑**G)** The results of neurobehavioral tests, including the Zea Longa score, forelimb slip test, screen test, and hanging test (n = 8). **(H)** The Nissl-stained sections of the hippocampal CA1 region and cortex (red arrows: damaged neurons; black arrows: normal neurons). **(I**‑**L)** The serum levels of IL-10, IL-4, TNF-α, and IL-1β measured by ELISA (n = 6). All quantitative data are expressed as mean ± SD. Scale bars: 50 μm (20×) and 20 μm (40×). ^##^ *p* < 0.01 vs. WT-sham; ^###^ *p* < 0.001 vs. WT-sham; ^**^ *p* < 0.01 vs. WT-model; ^***^ *p* < 0.01 vs. WT-model.

Nissl staining revealed severe neuronal damage in the cerebral cortex and hippocampal CA1 region of both WT and CKLF1−/− ischemic rats, including condensed Nissl bodies and nuclear pyknosis (Figure 7H). NGR1 partially restored neuronal morphology in WT rats but exerted no histological protective effects in CKLF1−/− animals, confirming CKLF1 is required for NGR1-mediated neuroprotection. ELISA results demonstrated lower levels of pro-inflammatory cytokines (IL-1β, TNF-α) and higher anti-inflammatory cytokines (IL-10, IL-4) in ischemic CKLF1−/− rats compared with WT controls (Figures 7I–L). NGR1 markedly suppressed IL-1β and TNF-α expression in WT rats yet exerted no regulatory effects on these inflammatory factors in CKLF1−/− rats, indicating CKLF1 is an essential intermediate for the anti-inflammatory activity of NGR1.

Collectively, loss of CKLF1 completely abrogated all therapeutic benefits of NGR1, including its improvements in cerebral edema, infarct volume and neurological deficits. These data validate that NGR1 alleviates ischemic brain injury by inhibiting neuroinflammation and neuronal apoptosis in a CKLF1-dependent manner. This work identifies CKLF1 as the direct molecular target through which NGR1 confers anti-ischemic neuroprotection, and the absent therapeutic response in CKLF1−/− rats demonstrates the indispensable role of CKLF1, laying a mechanistic basis for developing NGR1-based therapies for ischemic stroke.

To clarify whether CKLF1 mediates NGR1-regulated neuronal metabolic reprogramming, we detected the protein levels of CKLF1, CCR5, IL-1β, AMPK, mTOR, HIF-1α, PFKFB3 and GLUT3 in the cerebral cortex and hippocampus of wild-type (WT) and CKLF1−/− rats after pMCAO via WB. After cerebral ischemia, both WT and CKLF1−/− rats displayed similar pathological molecular changes: upregulated CCR5, IL-1β, phosphorylated mTOR, HIF-1α, PFKFB3 and GLUT3, accompanied by impaired AMPK activation (Figures 8A–V). In WT rats, NGR1 (40 mg/kg) reversed all these ischemia-triggered abnormalities, recovering AMPK activity and normalizing mTOR/HIF-1α signaling as well as glycolytic proteins. However, NGR1 exerted no corrective effects on these molecular dysregulations in CKLF1−/− rats, whose protein expression patterns were identical to vehicle-treated CKLF1−/− pMCAO rats. Collectively, these mechanistic data confirm that CKLF1 is an obligate molecular target required for NGR1 to exert neuroprotective and metabolic regulatory functions against ischemic brain injury.

**FIGURE 8:**
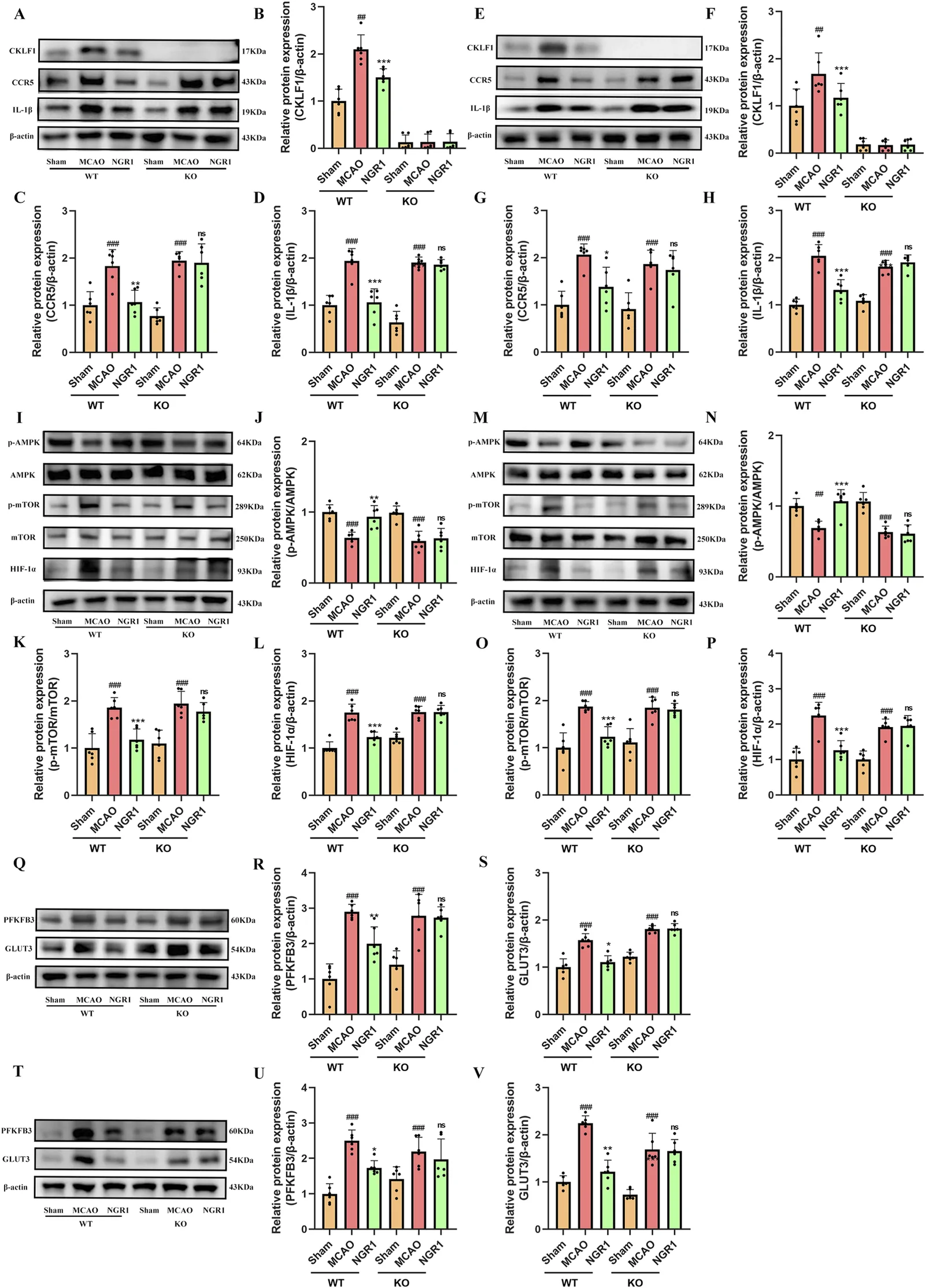
Knockdown of the CKLF1 gene prevents NGR1 from regulating neuronal metabolic reprogramming for the treatment of ischemic stroke. **(A**‑**H)** The results and analyses of WB determination of the hippocampal and cortical CKLF1/CCR5 axis with IL-1β protein expression. **(I**‑**P)** The results and analyses of WB determination of hippocampal and cortical AMPK/mTOR/HIF-1α signaling pathway expression. **(Q**‑**V)** The results and analyses of WB determination of hippocampal and cortical PFKFB3 and GLUT3 protein expression. All data results were expressed as mean ± SD (n = 6). ^##^ *p* < 0.01 vs. WT-sham; ^###^ *p* < 0.001 vs. WT-sham; ^**^ *p* < 0.01 vs. WT-model; ^***^ *p* < 0.01 vs. WT-model.

## 4 Discussion

Ischemic stroke arises from interrupted cerebral perfusion and initiates a vicious cascade of pathological events, including robust neuroinflammation, irreversible neuronal loss and cerebral infarction (Shin et al., 2020; Jurcau and Simion, 2021). Insufficient oxygen and glucose supply disrupts cerebral energy homeostasis. Mature neurons mainly rely on oxidative phosphorylation to produce ATP, rendering them highly vulnerable to energy collapse under ischemic conditions (Bolaños and Almeida, 2010). Rapid ATP exhaustion after ischemia destroys ion balance and activates multiple damaging signaling cascades, further aggravating brain injury (Suarez, 2012). Therapeutic strategies targeting metabolic reprogramming and neuroprotection are urgently needed to limit ischemic damage. Sanchi, a well-known traditional Chinese medicinal herb with properties of activating blood circulation to dissipate stasis, has been widely used for stroke with blood stasis syndrome. As a characteristic active saponin from Sanchi, NGR1 exhibits promising therapeutic potential against ischemic stroke (Yang et al., 2024; Zeng et al., 2026). Consistent with prior pharmacokinetic findings, NGR1 can penetrate the blood-brain barrier and distributes widely across multiple brain regions, including the cerebral cortex, hippocampus and striatum (Guo et al., 2014). In this work, we adopted the pMCAO model, which closely recapitulates pathological features of clinical thromboembolic stroke, and validated the neuroprotective efficacy of intraperitoneally administered NGR1, supporting its translational prospect for critically ill stroke patients.

CKLF1 is increasingly recognized as a critical therapeutic target for ischemic brain injury (Kong et al., 2014; Chen et al., 2019; Zhou et al., 2022). Previous studies indicated that CKLF1 participates in regulating metabolic reprogramming and mitophagy dysfunction via the AMPK/mTOR/HIF-1α cascade (Ma et al., 2023; Wang et al., 2024); Analogously, glycolytic shift occurring in activated monocytes relies on the Akt/mTOR/HIF-1α signaling axis (Cheng et al., 2014). Nevertheless, whether CKLF1 disturbs neuronal glucose metabolism and energy homeostasis under ischemic stress remains largely uncharacterized. Herein, our results reveal that NGR1 ameliorates ischemic neuronal damage by interfering with the CKLF1/CCR5 axis, providing new mechanistic evidence linking metabolic reprogramming to stroke intervention.

The current study identifies CKLF1 as an endogenous mediator driving ischemic pathological progression. Multiple target-verification assays confirm that NGR1 directly interacts with CKLF1 and disrupts the CKLF1-CCR5 complex, further suppressing aberrant AMPK/mTOR/HIF-1α-dependent metabolic reprogramming. These observations extend the biological function of CKLF1 beyond its well-documented pro-inflammatory activity. We propose that the CKLF1-CCR5 complex acts as a membrane-associated metabolic checkpoint that accelerates neuronal energy collapse in the acute phase of cerebral ischemia. Notably, although Co-IP verified intracellular interaction between CKLF1 and CCR5, further experiments are required to clarify whether NGR1 interferes with extracellular ligand-receptor binding at the cell membrane (Figure 9).

**FIGURE 9:**
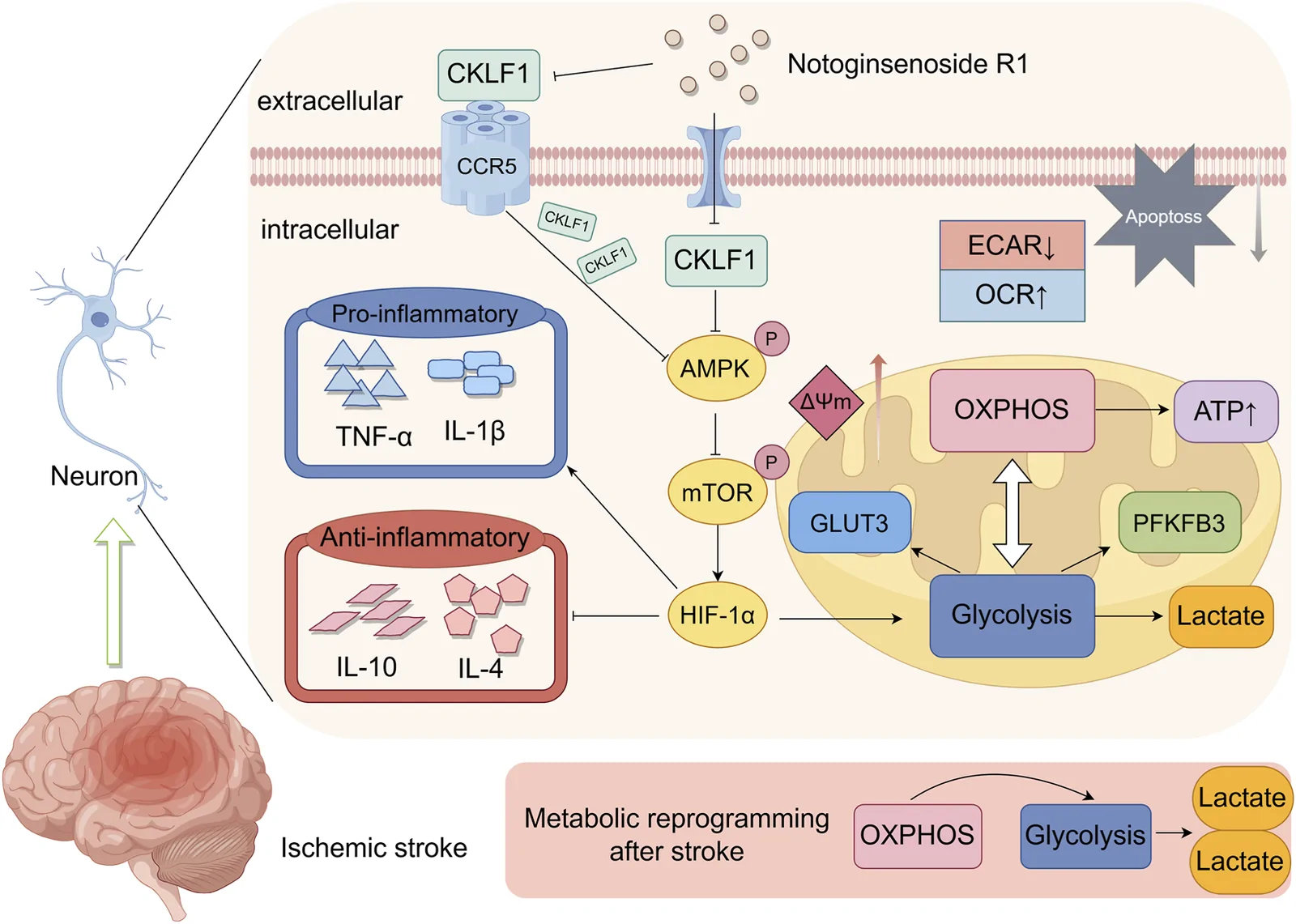
The mechanism by which NGR1 treats ischemic stroke involves regulating neuronal metabolic reprogramming induced by the CKLF1/CCR5 axis. After ischemic stroke, neuronal cells secrete CKLF1, which activates metabolic reprogramming by interacting with its receptor CCR5 in the AMPK/mTOR/HIF-1α pathway, converting neuronal metabolism from oxidative phosphorylation to the glycolytic pathway, increasing the flux of glycolysis, increasing the expression of PFKFB3 and GLUT3 proteins, generating neuroinflammatory responses, promoting lactate secretion, and inhibiting ATP production, forcing neurons to undergo apoptosis and neuronal death. Inhibit ATP production, forcing neurons to undergo apoptosis and neuronal death in large numbers. NGR1 treats ischemic stroke by interfering with the CKLF1/CCR5 axis and inhibiting neuronal metabolic reprogramming, reducing glycolytic flux, decreasing lactate production, promoting ATP release, and inhibiting peripheral inflammatory responses (By Figdraw).

Mechanistically, under ischemic/hypoxic conditions, neurons shift from oxidative phosphorylation to glycolysis. OGD reduces OCR and ATP levels while increasing ECAR, LDH, and lactate levels; these changes are reversed by NGR1 treatment. Activation of the CKLF1/CCR5 axis inhibits AMPK phosphorylation and aberrantly activates mTOR, leading to HIF-1α stabilization. HIF-1α upregulates glycolytic enzymes and glucose transporters, establishing an “ischemic glycolytic switch,” which subsequently causes ATP depletion, lactate accumulation, intracellular acidification, loss of mitochondrial membrane potential, and ultimately induces apoptosis. We define this cascade as “CKLF1-driven metabolic reprogramming-dependent neuronal death.”

Notably, NGR1 also alleviates peripheral inflammation and apoptosis, but these are downstream consequences of metabolic correction. HIF-1α-driven hyperglycolysis promotes the release of IL-1β and TNF-α through activation of pro-inflammatory signaling, while mitochondrial dysfunction directly activates endogenous apoptotic pathways. By correcting the upstream metabolic dysregulation mediated by the CKLF1/CCR5 axis, NGR1 achieves pleiotropic neuroprotection: restoring ATP production, alleviating lactate toxicity, suppressing the release of inflammatory mediators, and blocking apoptotic signaling. Thus, the CKLF1/CCR5 axis operates upstream of metabolic decision-making, and cellular metabolism fundamentally governs downstream functions, including inflammatory responses and the determination of cell survival versus death. An apparent discrepancy was observed in CKLF1−/− rats: although CKLF1 deficiency reduced ischemic injury, ischemia still activated AMPK/mTOR/HIF-1α and glycolytic proteins to a similar extent as in WT rats. This suggests that CKLF1 is required for the protective action of NGR1 but is not the sole upstream driver of ischemia-induced metabolic reprogramming. Other ischemia-sensitive pathways may activate AMPK/mTOR/HIF-1α independently of CKLF1. Therefore, the CKLF1/CCR5 axis should be viewed as a critical permissive node for NGR1-mediated metabolic correction rather than the exclusive initiator of post-ischemic glycolysis.

Neuroinflammation and neuronal metabolic dysfunction are likely linked bidirectionally rather than through a strict unidirectional cascade. Although our data place CKLF1/CCR5-AMPK/mTOR/HIF-1α upstream of glycolytic reprogramming and inflammatory cytokine release, inflammatory mediators can in turn impair mitochondrial function and amplify metabolic stress (Mitra et al., 2022). Thus, the present findings support a feed-forward pathological loop in which CKLF1/CCR5 signaling contributes to, and is potentially reinforced by, neuroinflammation. AMPK is not merely a downstream node of CKLF1/CCR5 signaling; it is a central energy sensor that regulates mitochondrial homeostasis, autophagy/mitophagy, glucose uptake, and neuronal survival (Li et al., 2025). In ischemic neurons, AMPK activity can exert context-dependent protective or detrimental effects. In our study, NGR1 restored AMPK phosphorylation and suppressed mTOR/HIF-1α-driven glycolysis, consistent with a metabolic switch toward energy homeostasis. Future work should determine whether AMPK activation is necessary and sufficient for NGR1-mediated protection.

From a translational perspective, the NGR1-CKLF1 metabolic intervention strategy offers distinct clinical advantages. Current gold-standard reperfusion therapies for ischemic stroke—rtPA thrombolysis and endovascular thrombectomy—are restricted by a narrow therapeutic time window (typically <4.5–6 h) (Moretti et al., 2015). In contrast, neuronal metabolic reprogramming is a pathological process that evolves over hours to days, suggesting the potential for a substantially extended intervention window. Post-ischemic NGR1 improved short-term outcomes in pMCAO rats; however, this preclinical finding does not establish clinical benefit beyond the reperfusion window. (Walie et al., 2026). Moreover, the mechanism of action of NGR1 is fundamentally distinct from that of conventional CCR5 antagonists such as maraviroc. Rather than directly occupying the ligand-binding pocket of CCR5, NGR1 binds to CKLF1 and disrupts its recognition and engagement with CCR5, thereby operating through a “ligand interception” mechanism. This approach may preserve physiological signaling between CCR5 and other endogenous ligands (e.g., MIP-1α), potentially avoiding the immunological side effects associated with complete chemokine axis blockade. Beyond the contrast with CCR5 antagonists, NGR1 also differs conceptually from traditional neuroprotective strategies that adopt a “damage antagonism” model—exemplified by antioxidants that scavenge reactive oxygen species, anti-inflammatory agents that suppress microglial activation, and anti-apoptotic drugs that block caspase cascades (Moretti et al., 2015; Liu Y. et al., 2022; Xu et al., 2022). These approaches merely counteract downstream injury events without rectifying the underlying metabolic crisis. By contrast, NGR1 actively remodels intrinsic neuronal energy metabolism through CKLF1-mediated metabolic reprogramming, thereby enhancing neuronal resilience to the ischemic hypoperfusion microenvironment.

While the present study provides robust multi-level evidence, several limitations should be acknowledged and may inform future investigations. First, the pharmacokinetic profile of NGR1—including its bioavailability and metabolic fate in vivo—remains to be fully characterized. Second, PC12 cells are a neuronal-like cell line and, even after differentiation, cannot fully recapitulate the metabolic phenotype of primary mature neurons. Therefore, the present *in vitro* findings should be interpreted as neuronal-like responses and require validation in primary neurons. Third, the use of whole-brain homogenates precludes rigorous discrimination of cell-type-specific metabolic alterations, thereby limiting precise characterization of the neuronal metabolic signature. Addressing these gaps will refine the mechanistic understanding of NGR1-targeted CKLF1 therapy for ischemic stroke and facilitate its eventual clinical translation.

## 5 Conclusion

This study demonstrates that NGR1 exerts neuroprotection by targeting CKLF1, thereby rectifying ischemia-induced metabolic dysregulation and suppressing peripheral inflammatory cascades. Mechanistically, we show that the CKLF1/CCR5 axis drives pathological neuronal metabolic reprogramming—characterized by enhanced glycolysis—through modulation of the AMPK/mTOR/HIF-1α signaling pathway, and that NGR1, by disrupting CKLF1-CCR5 binding, inhibits this reprogramming, reduces neuronal apoptosis, and limits hypoxic-ischemic brain injury. These findings establish CKLF1 as a promising therapeutic target, provide novel mechanistic insights into post-ischemic neuronal death, and deepen our understanding of the neuroprotective actions of NGR1 in ischemic stroke.

## Data Availability

The original contributions presented in the study are included in the article/Supplementary Material, further inquiries can be directed to the corresponding authors.
